# Abandonment of dogs in Latin America: Strategies and ideas

**DOI:** 10.14202/vetworld.2021.2371-2379

**Published:** 2021-09-13

**Authors:** Daniel Mota-Rojas, Néstor Calderón-Maldonado, Karina Lezama-García, Leonardo Sepiurka, Rita de Cassia Maria Garcia

**Affiliations:** 1 Neurophysiology, Behavior, and Animal Welfare Assessment, DPAA, Universidad Autónoma Metropolitana, Xochimilco Campus, 04960, Mexico City, Mexico; 2 Ethology, Bioethics and Animal Welfare, Universidad La Salle, Colombia; 3 Specialist in Canine and Feline Clinic of the College of Veterinarians of the Province of Buenos Aires. Small Animal Traumatology Specialist Professional Council CABA, Argentina; 4 Veterinary Medicine of the Collective and Legal Veterinary Medicine, Federal University of Paraná, Brazil.

**Keywords:** canine overpopulation, dog population management, free-roaming dogs, public health, stray dogs, zoonosis

## Abstract

In this article, we gathered information from postgraduate theses and scientific articles published in several databases using inclusion criteria that had been made in Latin America, in countries with similar economic conditions, and also in the USA to present a point of comparison. The objective of this review is to broaden the readers’ understanding of the causes of the increasing numbers of stray dogs and the reasons why people abandon pets in the streets, specifically in Latin America. It also discusses adoption and responsible ownership, identifies what failed in promoting positive human-dog interaction, and suggests strategies to address this problem. It concludes that adoption alone is not an effective solution but that it is necessary to offer education and awareness programs for owners, organize sterilization campaigns, and develop and apply – with the corresponding authorities – measures to ensure animal welfare that will provide benefits for society and improve animal quality of life. The role of veterinarians is fundamental in education and in disseminating the necessary information to orient people before they acquire a pet and prevent animal abandonment to resolve this problem.

## Introduction

**Figure-1:**
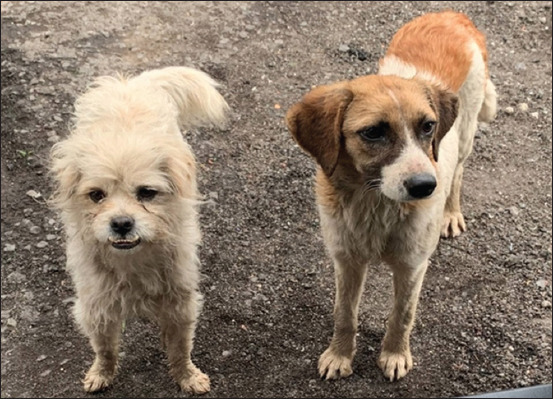
Free-roaming dogs.

Stray dogs are those that do not have a home but live on the streets [1]. According to the International Companion Animal Management Coalition [2], different forms of canine roaming include roaming dogs, free-roaming dogs, free-ranging dogs, stray dogs, and community dogs. For example, free-roaming dogs (Figure-1) are not under the direct control of a human. They may have owners but are allowed free access to the streets for certain periods of time or throughout the day. In other words, they have no defined physical barriers [3]. Another type is the so-called community dog, one that may have various owners. Of the 500,000,000 dogs in the world, 75% are stray dogs or offspring from uncontrolled breeding and human negligence [4].

Globally, stray dogs tend to be a disturbance that causes serious health, political, socioeconomic, and welfare problems [4-9], particularly in developing countries [10]. These problems include roaming the streets (causing traffic accidents) [11], barking (especially at night), aggression [12,13], and biting [14,15].

With regard to health, some reports indicated that 60-95% of all animal bites are caused by dogs in urban areas from Puerto Rico and Brazil [16-18]. In some cases, dogs can become vectors for transmissible diseases due to their close interaction with humans [12,19,20]. Moreover, they generate environmental pollution [21] by ripping open trash bags [22] and defecating and urinating in public. Although not all stray dogs are large in size, a large dog can defecate around 340 g/day, so fecal contamination can become a significant public health issue [19]. For example, in the city of Quito, they have approximately 150,000 stray dogs, in which pet owners do not properly dispose of their pets’ feces, the city’s garbage collecting services are not very efficient, and potentially the sewerage system receives around 51 tons of droppings per day [23]. Moreover, dogs excrete around 20-100 mL of urine per kg of body weight per day. Assuming that the average weight of a stray dog is 12 kg and that it produces 60 mL of urine per kg of weight per day, researchers estimated that each dog would excrete 720 mL of urine. This opens a window for the transmission of diseases to humans and contamination to the environment [19,24].

As in many Latin American countries, the control of free-roaming animals in public areas in Argentina is a problem that increases continuously. In 2014, a socio-environmental emergency was declared in Tierra del Fuego due to the presence of feral dogs in periurban areas and free-roaming dogs in cities. In Córdoba, government authorities were being urged to take measures to address the number of cases of people who were attacked by aggressive dogs in public [25]. Another facet of the problem is that stray dogs cause traffic accidents [6,24,26]. Moreover, free-ranging dogs can cause animal losses in small-scale farms [27]. From the perspective of public health [28], it is important to understand that dogs can be affected by over 100 zoonotic, bacterial [29], viral [30], and parasitic diseases [31-33] and may be carriers of diseases that include rabies [34-37], leptospirosis [38], hookworm disease, echinococcosis, leishmaniasis [39], ehrlichiosis, anaplasmosis, brucellosis [40], dirofilariasis [41], *Bartonella* spp. [42], cestodiasis, salmonellosis, campylobacteriosis, yersiniosis, helicobacter, *Bordetella pertussis*, *Borrelia burgdorferi* [43], and streptococcus, as well as staph infections, chlamydia, and scabies, among others [44,45]. Diseases like rabies exhibit a huge impact in some countries in Asia (China, India) and Africa, wherein studies identified 55,000 human deaths annually as a consequence of this disease. If we compare these figures with their total population, they may not be so large. However, in other countries, diseases, such as rabies, have been eradicated and further research is important [45]. Besides, rabies is a fatal disease that is present in almost every continent, but more than 95% of human deaths associated with rabies occur in Africa and Asia [46]. Furthermore, dogs are the reservoir of rabies diseases in many developing countries, such as Africa, India, and Southeast Asia [47-50].

**Figure-2:**
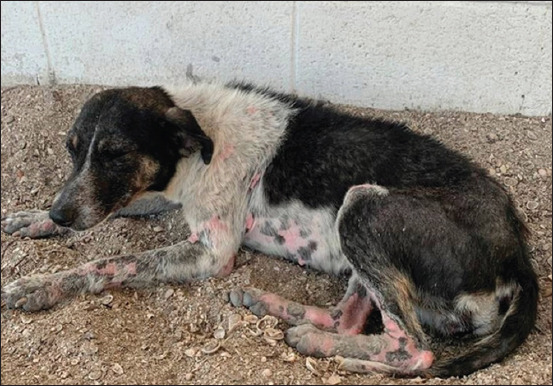
A stray dog with severe skin issues caused by demodicosis (Scabies). Note the areas with erythema and alopecia.

The fact that dogs are prolific compounds these problems. In 6 years, one female dog and her offspring exhibit the capacity to produce 67,000 new puppies [47]. However, according to Ibarra *et al*. [26] and Morales *et al*. [48], it must be considered that a large part of stray dogs do not reach reproductive ages due to all the risks they experience every day, because they demonstrate very low levels of welfare and quality of life due to the danger of being ran over, poor nutrition, exposure to disease, lack of shelter, and uncontrolled reproduction, among other factors [26,48] (Figure-2).

Dog abandonment has been identified as one of the main problems of animal population management [51,52]. One key question centers on the causes of the abandonment of these animals. According to Cendón *et al*. [53], the main factors include lack of space, lack of economic solvency to cover the expenses entailed in keeping pets, owners who tire of the disturbances that pets cause (aggressiveness, destructiveness, inappropriate elimination, barking, and roaming) [54,55], the fact that pets may cease to be a “novelty,” animals that go out of fashion, or the acquisition of another pet [56]. However, Cendón *et al*. [53] concluded that the main causes of abandonment are irresponsibility, lack of consciousness, and poor ethics of the owners. Therefore, the objective of this review is to broaden our understanding of the causes of the increase in the number of stray dogs, specifically in Latin America, while also discussing the concepts of adoption and responsible ownership, analyzing what failed in the human-dog interaction, and suggesting strategies to address this problem.

## Causes of the Increase in Stray Dogs and their Population in Latin America

The increasing population of stray dogs in Latin American countries is alarming (Table-1) [1,4,55-68]. In fact, specialists [57] are concerned that this problem is becoming increasingly difficult to resolve. For example, the study by Rendón *et al*. [57] in Peru found a ratio of 1 dog for every 3.98 people.

**Table 1:** The human and stray dog population in in selected countries of Latin America.

Country	Human population	Stray dog population
Chile	19’107,000 [58]	214,933 [55,59]
Brasil (Sao Paulo)	12.176.866 [55,60]	1000 dogs/km^2^ [61]
Ecuador	17.268.000 [62]	120,00 [56,59]
Colombia	50.374.000 [63]	4’224,575 [64]
Mexico	64’540,634 [65]	16’100,000 [1,66]
Uruguay	3.461.734 [67]	800,000 [4]
Peru	32.971.846 [57]	6’000,000 [68]

Abandonment is, without doubt, the main cause of the huge number of dogs that roam the streets or live in canine shelters [69]. While various factors contribute to the increase of their population, the most common one is abandonment by owners who realized that keeping a dog is not what they expected, due to the commitment that comes with being the guardian of a pet as well as the need to feed, bathe, and train them [47] or because they moved to a smaller home. But economic aspects [70], lack of time to care for them, management of vacation time, the disturbances they may cause in the neighborhood, the fact that puppies grow, and the realities of sickness and old age are all contributing factors. The study by Santos [55] identified and measured the following causes of abandonment: Aggressiveness (83.6%), sickness (38%), behavioral problems (20.9%), moving to another home (13.3%), lack of space (3.8%), pregnancy (3.8%), causing problems (3.8%), nearing death (3.8%), and old age (3.8%).

According to a research by Patronek *et al*. [71], most dogs abandoned in shelters are less than 6 months or over 8 years old. Some were acquired as presents, but with no previous consultation with the receiving family, and many were reproduced without control or responsibility due to the absence of a culture of sterilization or health problems that affected the owner or his/her family [53]. Another important cause of abandonment or of leaving pets in shelters [72] was problematic behavior [73,74], especially aggressiveness [54,75,76], but also hyperactivity, destructiveness, inadequate defecation, and excessive barking [77,78]. A study conducted in Spain showed that 91% of the animals left in shelters are found by civilians or the police in public areas. The other 9% are brought in by owners who abandon them due to undesired litters (15%); the end of the hunting season (12%); economic factors (12%); behavioral problems (11%); and loss of interest (10%) [79]. Despite the fact that these studies were conducted in the USA and Spain, they give us an idea that the causes of abandonment are similar in the rest of the world, including Latin America.

**Figure-3:**
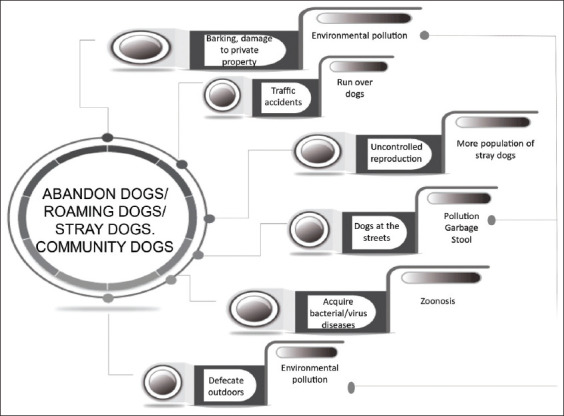
Adverse factors of human abandonment and free-rooming dogs for public health. Problems begin with the lack of responsibility of owners who abandon their pets in the street and finally, it could cause all the problems mentioned at the figure.

Another aspect of dog abandonment is that some owners refuse to practice responsible ownership, simply allowing their dogs to roam freely, alone in the streets where they cause many of the same problems as stray dogs [26]. Furthermore, because of their access to the streets, these dogs are free to reproduce, so they contribute to the increasing population of stray dogs [47]. Moreover, most of the time, pet owners due to their ignorance compound the problem when they think they must reproduce their pets to avoid possible psychological trauma [80]. Pets may also be abandoned by their owners due to the emergence of negative interactions with them, a factor that greatly increases the population of stray dogs and impacts the quality of life of the residents of the affected area [81,82] (Figure-3). It should also be considered that in many countries in Latin America and in some countries of the Orient, such as Taiwan and Japan, sterilizing pets is not as popular as in other nations. In Taiwan, for example, only 20% of pets are sterilized [76], while the figure for Japan is even lower than 12% [83]. These figures contrast strikingly with those of the USA, where 70% of pets are sterilized [84]. When we add to all this evidence the fact that the populations of many Latin American countries, including Mexico, suffer from extensive poverty (in the first quarter of 2021, on average, the extreme poverty lines by income (monetary value of the food basket) increased annually by 3.7% in urban areas and 4.0% in rural areas) [80] – a condition that often goes together with violence and the mistreatment of animals – then we can understand that the topic of animal welfare is of only secondary importance. Finally, it is clear that some people acquire companion animals without being duly prepared, for they lack knowledge, education, and/or sensitivity regarding them. Once they realize they cannot manage the responsibility that comes with acquiring a pet, abandonment surfaces as the expedient immediate solution [1].

## Strategies and Ideas to Address the Problem

Since the decade of the 1970s, many countries implemented programs that were based initially on eradicating animals but gradually shifted their focus toward controlling reproduction, education on responsible ownership, legislation, and animal registration [6]. Countries such as Spain exhibit codes and regulations governing responsible pet ownership, which establish that people must do all that is necessary to ensure animal welfare, including satisfying their needs for food, space, hygiene, vaccinations, and parasite control; sterilization; providing a device that clearly identifies the owner to impede dogs from roaming without control; and, in some cases, registering the animal in a centralized database if the legislation stipulates it [53]. A law against animal mistreatment came into effect in Mexico City on February 1, 2013, and campaigns have been conducted to increase the awareness and education of pet owners. One program implemented by the Ministry of Health of Spain is called “Be a responsible owner” (*Sé un dueño responsable*) [1]. However, in some Latin American countries, like Mexico, initiatives of this kind do not exist. Worse yet, an oversupply of dogs can be observed due to the operations of both professional and backyard breeders. This is a complex problem that requires authorities in every city to apply strategies to effectively reduce the birth of new puppies. Anti-rabies vaccination campaigns will be insufficient as long as the population of the new puppies are not controlled. Many countries opted to implement sterilization campaigns, programs to eradicate abandoned dogs, and educational initiatives [69], but neither eradication campaigns [47] nor sterilization programs have been proven effective [82] due to the quite common refusal of male owners (superior than females) to castrate male dogs [22]. Promoting the adoption of abandoned animals could be a viable solution but only if potential owners are obliged to sign an adoption contract where they agree to sterilize the pet, provide identification, and give all the required vaccines [53].

## Adoption versus Responsible Ownership?

So, is adoption a solution to the problem of stray dogs in the streets? We think that it is certainly not a bad idea, but not all stray dogs are suitable for reinstatement in a home due primarily to behavioral problems. The most common behavioral problems reported by owners as the reason for abandoning their dogs in shelters are aggressiveness toward people, dirtying the house, destructiveness, attempts to escape [54], hyperactivity, and barking. A study by Wells and Hepper [85] determined that the main behavioral problems during the 1^st^ month after adoption were hyperactivity and difficulties in socializing, followed by destructiveness, inadequate defecation, roaming, and coprophagy.

Another important aspect of the problem is that the dogs that are not adopted from shelters are euthanized. Researchers estimated that of 6–8 million dogs abandoned in shelters annually, half are euthanized (3-4 million) [71,86]. The environment of shelters is often an extremely stressful factor for dogs due to the excessive noise (the barking of other dogs), the need to modify habits, being locked up, and changes in light/dark cycles. The result of these elements is that shelters generate behavioral changes in the animals, so when they are adopted and arrive at their new home, they often show many behavioral problems that lead, once again, to abandonment [72,85].

The importance of shelters and the health of animals that live there has been emphasized in recent years due to the great ties that have been formed between humans and animals and because of the existence of zoonotic diseases. Thus, good shelter medicine programs are needed to maintain good overall health in animals as well as to prepare them for adoption [87-91].

In countries, such as Mexico, the topic of adoption is uncommon because of a culture that dictates that when you want something, you buy it. Responsible pet ownership is another uncommon topic, partly due to poverty and lack of education regarding the characteristics, needs, and care of animals. The basis for fostering respect toward animals is education, so, if no educational initiatives exist, they must be implemented. Moreover, people who fail to respect animals should be punished [1].

## The Human-dog Interaction: Why it Fails to Function

According to Said [1], the dog formed part of our lives since Neolithic times, participating in work around the home, pulling sleds, accompanying hunters, and helping people care for their homes and other animals. It was not until the Middle Ages that dogs became social symbols that give people prestige [1]. Gradually over time, stronger links have been forged with these animals. Due to new family structures, humans came to understand that dogs bring psychosocial benefits by forming part of the family nucleus, filling in for missing family members, and showing their affection [53,64,92,93]. Through their interactions with people, dogs perform various functions: from shepherding animals and hunting to offering protection and vigilance, pulling or carrying loads, and even accompanying their owners during diverse types of physical and psychological therapies and social problems [94]. A study by Valsecchi *et al*. [95] concluded that after 60 days of interacting with a human (playing, petting, and obedience training), dogs achieved better results on a temperament test that assessed socialization and obedience, compared with control dogs that did not have such interaction. In a laboratory study of dogs, Hubrecht [96] found that during the daily period of time with human interaction, dogs spent less time gnawing on objects around them. It is interesting to note that studies demonstrated that the gender of the person who interacts with a dog affects its behavior, as these animals tend to adopt a more defensive stance in the presence of men than women [97]. A study by González and Landero [98] found that 57.1% of their respondents considered their dogs to be members of the family, 31.2% viewed them as pets, and 11.7% saw them as guardians of the home. This suggests that the scope of dogs’ interactions with humans is widening [99].

Nonetheless, interaction between domestic dogs and humans can be affected by the appearance of behavioral problems related, primarily, to aggression, fear, separation anxiety, and, though to a lesser degree, compulsive disorders [100]. Thus, all the positive situations outlined above can be influenced by negative circumstances generated by people who acquire pets without doing any in-depth research on their characteristics and needs, with no prior analysis of the responsibility that comes with sharing the home with a pet whose life expectancy is 15 years and no assessment of the costs generated for food and veterinary care. In addition, researchers estimated that in countries, such as Quito, an average of 8567 dogs that are being ran over die every year [101]. Apart from being ran over, the use of poisons as a method of controlling populations faced with outbreaks of rabies causes severe suffering to animals, and it is due to these types of situations, together with sexually transmitted diseases, that the life expectancy of stray or free-roaming dogs is 3 years on average [23]. This is the point where the human-dog interaction fails, and irresponsible owners turn to the first option at hand: Abandoning their pets in the streets [1]. In contrast with Parry [102], with the recent COVID-19 pandemic, Morgan *et al*. [103] concluded that greater interest has been observed in the adoption of dogs and the percentage of abandonment in this period of social isolation did not change [103].

## The Role of the Veterinarian in Resolving this Problem

Veterinarians exhibit an important role to play in educating and informing owners about the basic needs of pets that, when satisfied, foster their welfare. This includes understanding the main aspects of the animal’s reproductive life. Convincing owners of the importance of preventing breeding is important by ensuring that they sterilize their pets as soon as possible [47]. In addition to decreasing the abandonment of dogs, sterilization helps decrease the risk of developing mammary tumors and uterine infections in females and testicular tumors in males [104]. The participation of veterinarians should center on the aspects of clinical medicine and animal health and nutrition, but they should also educate owners on pet management [64] and explain the relation between the mistreatment of animals and domestic violence. Several studies investigated the relationship between domestic violence and animal abuse [105-107,108-111]. Furthermore, veterinarians are inclined to detect this type of animal abuse, since they can exhibit direct contact with the animals that suffered from it [107]. Moreover, according to the study by Weng *et al*. [112], abandonment must be recognized as a form of mistreatment and many aspects of this problem are generated by disinformation, so the work of veterinarians is important and influential in educating owners about these topics because most people understand information better when they receive it from a health professional than in written form. Considering this when someone is contemplating acquiring a dog as a pet or as a working animal is particularly important for all concerned. Speaking with a trustworthy veterinarian is necessary for them in order to make the best choice, taking into account not only the place where the animal will live but also the age of the people with whom it will interact [113]. Thus, schools and local, regional, and national veterinary associations, as well as independent veterinarians, must strive to publicize these topics with regard to responsible pet ownership through multimedia channels and in conjunction with local governments, humanitarian societies, and citizen groups concerned with animal welfare. Only in this way will the message reach a much broader audience [114-120].

## Conclusion

The abandonment of dogs in the streets is a common situation in countries, such as Latin America. The causes of abandonment include the lack of education on responsible pet ownership; inadequacy or non-existence of measures of animal protection that impede the reproduction of dogs, while encouraging mandatory sterilization as a requirement for acquiring a pet; the lack of permanent sterilization campaigns; and extreme poverty in some communities and neighborhoods that keep both owners and their pets malnourished, among others. Conversely, several factors favored the increase of the number of pets, such as the following: Demand of pets to fill affectionate spaces in familiar environments, increased capacity economic status of social classes, allowing them to assume expenses previously not contemplated in your budget, and the phenomenon of displacement of peasant populations from rural areas, bringing with it the culture of owning animals. The problem now reached uncontrolled proportions, and this can be observed when going out on the streets and observing a large number of stray dogs in many developing countries. Moreover, this situation in Latin American countries tends to be considered normal or at least so common that it gets overlooked. Canine roaming exhibits a tendency to be normalized as a part of culture. We firmly believe that the most important of all the measures available to address this situation involves educating the population to raise awareness because, truth be told, none of the other strategies will be of much use if the main problem is not resolved. It is urgent that the competent government authorities become more involved in elaborating, improving, and applying laws for the protection of animals, where the obligations of pet owners are clearly stipulated and designed to improve the welfare of both animals and society as a whole. Moreover, the buying and selling of animals must be regulated, and the number of shelters for abandoned animals that encourage sterilization and opt for adoption over euthanasia must be increased. To complement this, people need to be made more aware through educational programs in which veterinarians, government officials, NGOs, and academic institutions, among other actors, participate because, as is more than clear, simply fomenting adoption does not solve the problem. Training veterinarians to detect behavioral problems in companion animals in a timely manner is also important, so that they are treated promptly and the animals do not end up abandoned. The government and citizens of each country, state, and city must work together to change this situation because the increase of canine roaming will not be solved by creating laws to protect animals that are rarely enforced or by exposing cases of animal mistreatment. Defining the pet owners’ rights and responsibilities and determining whether infringements of the law will lead to civil or penal responsibility is imperative. We must follow the example of several Latin American countries, such as Argentina or Colombia, that not only punish irresponsible pet owners but have gone much further by implementing training courses for people who decide to adopt a pet, supported with manuals, tutorials, and guides. Finally, credentials can be issued to identify owners who adopted in a responsible manner.

## Authors’ Contributions

DM: Conceptualized, drafted, and supervised the final version. DM, KL, NC, LS, and RCMG: Contributed to the original draft, data curation, investigation, writing, review, and editing of the manuscript. KL and DM: Worked on the methodology, writing, and editing of the review. All authors have read and approved the final manuscript.
